# Culturally congruent mentorship can reduce disruptive behavior among elementary school students: results from a pilot study

**DOI:** 10.1186/s40814-018-0339-8

**Published:** 2018-09-14

**Authors:** Arthur H. Owora, Najah Salaam, Sydney H. Russell Leed, Dessa Bergen-Cico, Timothy Jennings-Bey, Arnett Haygood El, Robert A. Rubinstein, Sandra D. Lane

**Affiliations:** 1 Department of Public Health, Falk College, Syracuse University, Syracuse, NY 13244 USA; 2 Street Addiction Inc., Syracuse, New York USA; 3 Upstate Medical University, Syracuse, USA; 4 Maxwell School, Syracuse University, Syracuse, USA

**Keywords:** Culturally congruent, Mentorship, Elementary school, School violence, Disruptive behavior

## Abstract

**Background:**

Our study objective was to examine the feasibility of implementing a culturally congruent mentorship pilot program, Youth-First (YF), that targets behavior modification among elementary school-aged children with disruptive behavior and a history of school suspension. We hypothesize that it is feasible to implement the YF program to reduce disruptive behaviors and recidivism of level III/IV infractions in school settings among at-risk African American students.

**Methods:**

We assessed program feasibility based on the success of program acceptance by parents/guardians, study enrollment, and intervention compliance by students. A pre/posttest study design was used to examine whether the YF program reduced recidivism of disruptive behavior among enrolled at-risk African American elementary school children between September 2016 and January 2017. Generalized linear mixed models examined whether student behavioral scores improved over time and varied by program mentor. A McNemar test examined the reduction in cumulative incidence of level III/IV infractions pre-post YF program intervention.

**Results:**

Intervention acceptance, enrollment, and compliance were 100% (95% confidence interval [CI] 86 to 100%), 100% (95% CI 86 to 100%), and 67% (95% CI 45 to 84%), respectively (*N* = 24). Overall, student behavioral scores improved and plateaued over time (Time^2^ effect: *b* = − 0.01, 95% CI − 0.02, < 0.01); a two-week period was associated with a seven-point improvement (effect size: Cohen’s *d* = 0.47, 95% CI 0.03, 0.94) in behavioral scores. Behavioral score improvements were class-specific, based on respectfulness behavior (*b* = 0.11, 95% CI < 0.01, 0.26). No recidivism of level III/IV infractions was reported during and post YF intervention.

**Conclusion:**

The integration of culturally congruent mentorship in elementary school-settings is feasible and can reduce risk of disruptive behaviors among at-risk African American students. Future studies should use randomized clinical trials to determine the effectiveness of culturally congruent mentorship interventions (void of potential selection and confounding biases) in reducing disruptive behavior, level III/IV infractions, and school suspensions among at-risk children.

## Background

Disruptive student behaviors that result in school suspensions are categorized as level III or IV infractions [1, 2]. Typically, level III or IV infractions involve the display of explosive, aggressive, or maladaptive patterns of behaviors by students that result in physical harm of other students and/or school personnel. Suspensions (in and out of school) are not always reserved for the most severe behaviors but also relatively minor offenses such as disobedience and disrespect, class disruption, and non-attendance [2]. Disruptive student behavior affects not only the immediate victims, but also spatially related classmates, parents, and school community [3, 4]. In a classroom, it negatively influences the learning process through loss of instructional time, loss of focus by other students, decreased student motivation, decreased student-teacher engagement, increased teacher stress, frustration, and in some cases attrition [3–5]. This underlines the extended reach of its impact regardless of the severity and frequency of the disruptive behavior.

The etiology of school-related disruptive behaviors is linked to early childhood behavioral and emotional disorders including but not limited to attention deficit hyperactivity, oppositional defiance, autism spectrum, depression, anxiety, bipolarity, learning, and conduct disorders [5, 6]. Although, more proximally related to elementary school-age children’s disruptive behaviors than behavioral and emotional disorders, sporadic and temporary adjustment problems have limited research [7, 8]. Yet, coupled with a lack of social trust in school environments, boredom, confusion, and resentfulness in a classroom setting can precipitate into classroom disruption [9]. To address some of these problems, interventions involving non-familial mentors may be critical for not only preventing class disruption that leads to school suspension but also ensuring prosocial success of elementary school-aged children through adolescence to adulthood [7, 8].

There is growing evidence showing that school mentorship programs that provide social support, social skills, emotional regulation, and problem solving skills lead to interconnected and sustained social-emotional, cognitive, and identity child development [8, 10, 11]. These programs have also demonstrated small-to-moderate effect sizes in the reduction of disruptive behavior [8, 10, 11]; however, their effect on level III/IV infractions that result in school suspensions is not well established.

Among elementary school children, the argument for preventing disruptive student behavior and consequently suspensions is compelling due to its adverse effects on the life course trajectory outcomes of students involved [12, 13]. This is especially true for minority and low-income students who tend to be at higher risk for school discipline-related referrals and are disproportionately over-represented in school discipline cases [14]. Disparities in school discipline and student academic outcomes due to racial and social economic status (SES)-related biases are well established [2, 4, 12, 13, 15].

Some studies suggest that the over-representation of minorities, especially African Americans, in school discipline incidents is due to cultural discontinuities that place them at a disadvantage in many public elementary schools [2, 4, 16]. Teachers, school personnel, and administrators in these schools, a majority of whom are of European origin, may be unfamiliar with the cultures, norms, and communication styles of minority students [16]. Minorities also tend to have lower SES backgrounds; however, minority race has been shown to be associated with disproportionately higher suspension rates independent of SES [4].

In Syracuse City School District (SCSD), one of the largest school districts in New York State with one of the highest cases of poverty within the nation; 85% of SCSD students are classified as economically disadvantaged [3]. Out-of-school suspensions are three-fold higher than in other school districts in the USA, and Black students are disproportionately affected compared to Whites (25% versus 12%) [3]. School-related violence affects not just students but the school system as a whole. Indeed, a survey conducted among 838 SCSD teachers in 2015 found that 66% were worried for their safety at work, 50% were harassed, 57% were threatened, and 36% were physically assaulted in school [17]. Teachers also indicated they did not feel prepared to handle violent situations (40%), had no access to a violence-prevention program (57%), and more than 50% felt the SCSD administration was uncommitted to violence prevention [17]. The magnitude of violence, victimization, and associated disruption in the SCSD severely impairs the educational process and the normal psychological development of students.

To address school-related violence, multi-faceted prevention strategies that address factors at multiple levels: students with problematic and disruptive behavior, relations with other students, teachers and school personnel, and community are needed [15]. Culturally congruent mentors can play an important role at the individual-student level and across these multiple levels. At the individual level, mentors can promote processes of autonomy, model desired behaviors, identify risk signs and modalities of disruptive behavior, as well as how these situations impact student learning and emotional well-being. Given their unique experience of related cultural, community, and societal context, mentors can mediate student engagement through dialogue/reflection, modeling desired behavior, and practice of social and coping skills. Mentors can also influence student-teacher/school personnel relationships by providing context and a better understanding of causes of disruptive behavior. Unfortunately, this approach to prevention of disruptive behavior and associated suspensions among at-risk minority students in school settings has limited research.

Based on the above considerations, this study aimed to examine the feasibility of implementing a pilot program, Youth-First (YF), that targets behavior modification among elementary school-aged children with disruptive behavior and a history of school suspension. The YF program specifically targets disruptive behavioral problems most proximal to school suspensions using an eco-behavioral perspective that involves a dynamic collaboration between parents, teachers, and culturally congruent community-based mentors. In this pilot study, we hypothesize that it is feasible (measured by program acceptance, enrollment, and compliance) to implement the YF program to reduce disruptive behaviors and recidivism of level III/IV infractions among at-risk African American students in elementary school settings.

## Methods

### Study design

Pre/posttest study design with no concurrent control group.

### Study setting

Elementary school X is located in Syracuse city in one of the highest gun clusters in New York State. Elementary school X has 650 students; 90% are of African-American or Hispanic decent; 93% participated in the National School Lunch Program, a federally assisted program for low-income families; and 22% have a diagnosed mental health disorder (e.g., emotional disturbance, attention deficit hyperactivity disorder, and oppositional defiant disorder). Elementary school X is one of the lowest performing elementary schools in Syracuse City; 98% and 95% of students performed below proficiency level on the New York State grades 3–8 English Language Arts and Mathematics assessments, respectively. The school also had a staff turnover of 33% during the 2013/2014 academic calendar.

### Study participants

Study inclusion criteria was based on receipt of three or more referrals for level III/IV infractions or failure to attend teacher referred school based behavior counseling between September 2016 and January 2017. We excluded children involved in school-based behavioral counseling.

### Study intervention

The pilot of YF program components included mentors: (a) acting as a first responder; (b) conducting daily Check-In Check-Out (CICO); and (c) helping to build relationships between student, teacher, and the student’s family. YF mentor caseloads varied between five to six students in any given week during the five-month intervention period.

- Acting as first responders

Teachers made calls to request for program mentors as first responders via a walkie-talkie device, if a student exhibits disruptive behavior in a classroom setting. Regardless of the infraction, a student is able to take a 15–30 min reset with their mentor to de-escalate before returning to class or before seeing an administrator. The de-escalation process involves practicing self-control and self-regulation techniques. Students also requested meetings with their mentor whenever they felt agitated during class. Prior to this accommodation, teachers requested the removal of a disruptive child from the class, often with the use of restraints.

b) Daily Check-In Check-Out (CICO)

Mentors conducted at least two daily CICO’s with each student (in addition to the first responder contact for those students who had a problem during the day) during the five-month intervention period. Mentors used CICOs as a means to engage with the student, gauge what type of day a student was having, and practice self-control and self-regulation techniques. Students earned prizes for scores above 27 on the CICO report. All students enrolled in the program also had lunch at least once a week in groups (based on grade level) with their program mentors (e.g., a group of seven students may have three third graders and four fourth graders). On average, each mentor dedicated between 30 and 45 min to each student each day depending on the number of student-initiated requests and/or their involvement in minor infractions (i.e., levels I and II infractions that do not result in school suspension).

c) Helping to build relationships between teacher and parents/guardians

#### Teacher relationships

In addition to interacting with assigned students, during the daily CICOs, program mentors observed and documented a student’s behavior during different class sessions (e.g., math, science) while focusing on specific behaviors (e.g., respect, stay in class, and accountability). Documented CICO reports included teacher perceptions. Program mentors provided teachers with a holistic context (school peers, family, and community) of each student’s well-being on a weekly basis. Mentors used interactions with the student’s teachers to inform mentor-student activities that supported student academic improvement and behavioral change in collaboration with an education consultant (NS).

#### Parental buy-in and trust

Each student’s parent/guardian received a permission slip explaining the purpose of the program, the objective of the program, the team’s community affiliation, and ways in which the mentor would work with the mentees during regular school hours. Program mentors also requested and received parental permission to work with the student on weekends during extracurricular activities (e.g., mentor/mentee spends a Saturday at a football game). Mentors made weekly calls to a student’s parent/guardian to update them on their child’s progress in the program.

### YF program mentors

YF program mentors were four community members (ages 34–50 years) who prior to the pilot intervention worked as gang outreach workers. Briefly, mentor previous experiences involved responding to every community murder or gunshot injury to provide tension/conflict reduction and emotional and self-regulation support to grieved individuals and first responders. The program mentors had histories of overcoming their own lived experiences directly related to neighborhood conflict. All program mentors had certifications in trauma informed and mindfulness-based interventions using self-control and self-regulation techniques.

### Primary outcome measures

The prevalence of program acceptance by parents/guardians, student enrollment, and intervention compliance by students was determined at the end of the study period (January 2017). Program acceptance by parents/guardians was determined based on the receipt of a signed permission slip consenting to a student’s program participation. The study threshold for successful enrollment was set at 90% for all eligible and contacted students and their parents/guardians. A student was considered intervention compliant if they had at least three training session contacts a month (for at least 15 contacts during the five-month intervention period) with their mentor (outside of first-responder and daily CICO contacts). During these meetings, mentors (a) explained the YF self-control and self-regulation techniques, (b) demonstrated techniques, (c) had students practice the techniques, and (d) provided students with corrective and positive feedback.

### Secondary outcome measures

Check-In Check-Out (CICO) measure: The CICO is a three-item scale that captures elementary school children’s behavior on three target domains: respectfulness, stay in class, and accountability. Respectfulness—focuses on how respectful the target child’s behavior is with adults and peers. Stay in class—documents the target child’s behavior in the classroom towards teachers and peers. Accountability—captures the target child’s perception of their problematic behavioral actions. A target child’s behavior on each of these three domains is rated on a three-point Likert scale of goals met (0—no goals met, 1—some goals met, 2—all goals met) across six periods (or classes): (1) math, (2) social studies, (3) specials, (4) recess, (5) language arts, and (6) science. For each domain and period/class (e.g., Respectfulness during Math class), three goals were determined on a weekly basis by the school administrator/teacher concerned (two goals) and the target child (one goal). Total possible scores on the CICO ranged from 0 to 36. For example, if a child met all goals on all three domains across the six periods, they received a total score of 36.

Level III/IV infractions: Level III behaviors involved (a) repeated incidents of level II infractions (e.g., swearing, electronic-based aggression including inappropriate social networking content, bullying, cyber-bullying, accessing inappropriate online content, cheating or plagiarism, possession or use of tobacco or alcohol or prohibited over-the-counter medication on school property); (b) behaviors targeted at or targeting others; and (c) behaviors that compromised individual or other students’ safety. Examples of level III behaviors included fighting, threats/intimidation, extortion, sexting, theft or vandalism [involving property less than $500], substance impairment, and propping open secured facilities including the school bus. Level IV behaviors involved (a) repeated incidents of level III infractions, and (b) behaviors that involve safety issues. Examples of level IV behaviors included suspected substance use or possession, physical assault, and theft/vandalism [involving property more than $500].

### Statistical methods

#### Sample size and power analysis

Given that this was a pilot study, we did not perform a sample size calculation for our primary outcomes (i.e., intervention—acceptance, enrollment, and compliance) a priori. We aimed for 24 students because it was felt that a caseload of 4–5 students per mentor (anticipating a response rate of 80%) was manageable and large enough to inform us about the practicalities of intervention delivery. Additionally, this sample size choice was supported by the sample size calculation for our secondary outcome—mean change in CICO behavioral scores. In order to detect a mean difference of nine points, pre-post YF program intervention, a sample size of 18 students was needed to have a power of 90% (at a type I error value of 0.05) assuming a correlation of 0.5 between repeated measurements and a standard deviation of 6 and 12 for pre-post CICO points. With 18 students, we had approximately 89% power to detect a paired proportion difference (based on a one-sided McNemar test) of 40% in III/IV infractions among students pre-post YF program intervention.

#### Statistical analysis

We calculated YF intervention acceptance, enrollment, and compliance prevalence (proportions) with 95% “exact” binomial confidence intervals. *Generalized linear mixed models* were used to examine changes in student CICO scores (total, behavior-specific, and period/class-specific) over time and whether these changes varied by program mentor. We modeled each student and behavior change (i.e., slope) as a random intercept and slope, respectively to account for individual level variation nested within mentor. Fixed time (including a quadratic term to capture potential plateau effects) and program mentor (including interaction terms) effects were estimated. A McNemar test examined whether the proportion of III/IV infractions decreased pre-post YF program intervention. We used SAS 9.4 (SAS Institute Inc., Cary, North Carolina) for data analysis, and all statistical tests were set at a .05 level of significance.

## Results

### Intervention acceptance, enrollment, and compliance

We met our study feasibility criteria goals with 100% (95% confidence interval [CI] 86 to 100%) YF intervention acceptance by parents/guardians approached for study consent. All targeted children (100%, 95% CI 86 to 100%) were successfully enrolled into YF intervention activities. Of the 24 enrolled students, 16 (67%, 95% CI 45 to 84%) were intervention compliant (i.e., had at least 15 contacts with a program mentor during the intervention period). Students had an average of 16 (95% CI 14 to 18) student/mentor contacts with a minimum of 10 and a maximum of 22 contacts. Program mentors received on average 20 (95% CI 17 to 23) calls per day that ranged from 5 to 10 contact minutes with students.

### Changes in CICO total scores

**Fig. 1:**
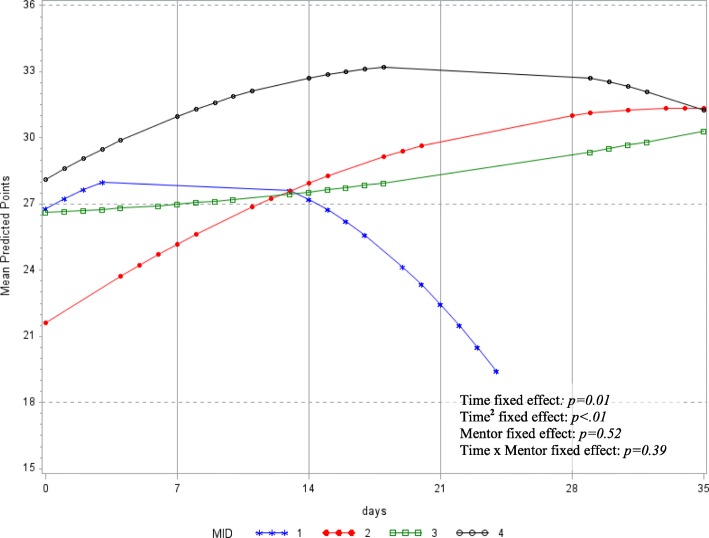
Predicted mean profile of student CICO total scores by program mentor (MID). Each symbol (star, square, filled circle, empty circle) and color represents the mean profile of CICO scores of students by program mentor (MID). Statistical model: CICO Points = *γ*_00_ + *γ*_01_(Mentor) + *γ*_10_(Time) + *γ*_11_(Time^2^) + *γ*_12_(Mentor*time) + *γ*_13_(Mentor*Time^2^) + [*u*_0j_ + *u*_1j_(Time) + *r*_ij_] − random component

Overall, student total CICO scores increased (i.e., improved) and plateaued over time (Time^2^ effect: *b* = − 0.01, 95% CI −0.02, < 0.00); a two-week period was associated with a seven-point increase (effect size: Cohen’s *d* = 0.47, 95% CI 0.03, 0.94) in CICO scores. Changes in student CICO scores did not differ by program mentor (Time × Mentor effect: Mentor _(1 vs. 4)_: *b =* − 0.43, 95% CI: − 0.73, − 0.13; Mentor _(2 vs. 4)_: *b =* 0.19, 95% CI − 0.02, 0.41; Mentor _(3 vs. 4)_: *b =* 0.06, 95% CI − 0.15, 0.27). Figure 1 shows the mean trajectory of student total CICO scores aggregated by program mentor improved over time for all but one mentor.

### Changes in CICO behavior sub-scales

Similar to total CICO scores, the respectfulness CICO sub-scale scores increased and plateaued over time (Time^2^ effect: *b =* − 0.01, 95% CI − 0.02, < 0.00) with no significant differences between program mentors (Mentor _(1 vs. 4)_: *b =* − 0.16, 95% CI − 0.24, 0.55; Mentor _(2 vs. 4)_: *b =* 0.10, 95% CI − 0.13, 0.32; Mentor _(3 vs. 4)_: *b = −* 0.09, 95% CI − 0.32, 0.13). The stay in class CICO sub-scale scores did not change over time (Time effect: *b =* 0.10, 95% CI < 0.01, 0.20) or between program mentors (Time × Mentor effect: Mentor _(1 vs. 4)_: *b =* − 0.06, 95% CI − 0.18, 0.07; Mentor _(2 vs. 4)_: *b =* 0.07, 95% CI − 0.01, 0.15; Mentor _(3 vs. 4)_: *b = −* 0.01, 95% CI: − 0.09, 0.07). In addition, accountability CICO sub-scale scores did not change over time (Time effect: *b =* 0.09, 95% CI − 0.02, 0.20; Time × Mentor effect: Mentor _(1 vs. 4)_: *b =* − 0.14, 95% CI − 0.27, − 0.01; Mentor _(2 vs. 4)_: *b =* 0.05, 95% CI − 0.04, 0.14; Mentor _(3 vs. 4)_: *b =* 0.01, 95% CI: − 0.07, 0.09).

### Changes in CICO class-specific sub-scales

Class specific CICO scores improved over time (with a plateau effect) for the math, language arts, and science classes (Time^2^ effects: all *b*_*s*_ *<* − 0.01). Changes in CICO score also differed by program mentors during language arts (Time × Mentor effect: Mentor _(1 vs. 4)_: *b =* − 0.09, 95% CI − 0.14, − 0.03; Mentor _(2 vs. 4)_: *b =* 0.04, 95% CI < 0.01, 0.09; Mentor _(3 vs. 4)_: *b <* 0.01, 95% CI − 0.04, 0.05) but not in other classes. During social studies classes, there was a marginal improvement in behavioral scores over time (Time effect: *b =* 0.02, 95% CI < 0.01, 0.03) but no improvements were observed during the special classes (Time effect: *b =* 0.03, 95% CI − 0.02, 0.09) and recess periods (Time effect: *b =* 0.02, 95% CI <− 0.01, 0.03).

### Incidence of level III/IV infractions

No recidivism of level III/IV infractions was reported during and post YF intervention.

## Discussion

Our pilot intervention results show that it is feasible (high acceptance, enrollment, and intervention compliance) to implement a culturally congruent mentorship program (YF) with demonstrable reductions in disruptive behaviors and recidivism of level III/IV infractions among at-risk African American students in elementary school settings. This is evidenced by behavioral score improvements that are class-specific (e.g., during math, language arts, and science class but not social studies, special, or recess class) and behavior-specific (i.e., respectfulness behavior) and did not vary by program mentor. During the five-month study period, none of the intervention students were involved in level III/IV infractions.

The acceptance of the YF program by parents/guardians is not new to this study and has been demonstrated before in other culturally congruent interventions that target school children [2, 5, 8]. What is new, however, are the high enrollment and compliance proportions by elementary school children. This to some extent may suggest that cultural congruence may be a critical component to engaging elementary school students. Specifically, the lived experience of mentors as minorities comes to bare and probably functions to calm students during critical time-points that define whether a situation escalates into disruptive behavior or de-escalates through self-regulation and control. Moreover, the fact that the mentors operate across a student’s spatial environments (class, home, and during extra curriculum activities) allows for a familiar non-threatening actor as a first-responder when a student relapses towards past disruptive tendencies.

In addition to cultural congruence, some of the successes in enrollment, acceptance, and compliance may be due to the YF program’s setting of clear daily CICO expectations, focus on building trust and friendship, mentorship training, and prior experiences mentors had in community trauma response. These factors have been associated with successful mentor-mentee relationships involving children in existing literature [8, 9].

Evidently, culturally competent mentors in collaboration with teachers and parents can intervene to decrease incidents of disruptive behaviors that result in school suspension. Pulling from their experiential vantage points, YF program mentors were able to identify the subtle signs, not obvious to parents/guardians, teachers, and school personnel, of impending disruptive behavior and instituted timely remedial measures to prevent follow-through by students involved. A mentor’s role is neither academic nor parental but engenders trust; cultural understanding; and accountability to a student, teacher, and parent. As such, a mentor has unique insights and rapport with students; this allows a student to confide in and build trust with him or her. As a function of a mentor’s exposure to the home, school, and community contexts experienced by a student, mentors can identify potential causes and conditions that lead up to disruptive behavior. With this knowledge, mentors can intervene, avert behavior escalation, and make referrals to address root causes of disruptive student behavior.

Variations in class-specific (versus behavior-specific) behavior improvements suggest a potential behavioral modification effect linked to conditions of a class involving specific teacher influences and to a lesser extent specific behaviors (e.g., accountability and stay in class). These teacher effects may be a function of teaching practices including but not limited to a teacher’s emotional support and classroom organization [18]. In fact, Pianta and Hamre (2009) postulate that emotional supports and organizational techniques are just as important as a teacher’s instructional methods in supporting student’s development beyond core academics [19]. Our findings, to some extent, suggest teacher effects may also extend to mentor effectiveness underlined by the mean differences in students’ behavior change between mentors during the language arts class but not in other classes. The potential for such teacher influences are consistent with existing research that show that some teachers have difficulty identifying risk indicators of disruptive students’ behavior, especially the more subtle signs that precede physical harm to themselves and other students [2, 4, 13]. Therefore, effective mentorship program interventions should not preclude strategies that target the improvement of the full range of teacher skills including those needed to improve student’s academic performance.

In the face of ever-growing teaching and student performance demands, it may be infeasible to expect teachers to shoulder the full responsibility of individual student behavior intervention. In this sense, collaboration with a mentor can promote preventive intervention that incorporates a broader holistic contextual understanding of a student’s family, school, and community environment and its influence on student behavior in school-settings.

It is important to acknowledge a number of limitations of our pre/posttest study design. One, we cannot conclusively attribute pre/post behavioral changes to YF intervention since we did not have a concurrent control group. For example, students given behavioral tests may have been inspired to behave better than those who are not tested independent of YF intervention effects. On the other hand, behavioral testing alone cannot explain the 100% reduction in level III/IV infractions among students with prior infractions, a known risk factor of recidivism [1, 6–9]. Two, the small study sample size (especially mentors: *n* = 4) did not allow for an examination of mentor characteristics that might have influenced their effectiveness. This and potential cross-level (student/mentor) factor interactions need to be examined in future studies.

## Conclusions

In summary, it is feasible to integrate culturally congruent mentorship in school-settings to reduce risk of disruptive behaviors among minority, African American students. Culturally congruent risk identification and intervention, as well as teacher training are needed to holistically address disruptive student behavior that result in school suspension and adverse downstream outcomes [13] for at-risk students. Future studies should use randomized clinical trials to determine the effectiveness of culturally congruent mentorship interventions (void of potential selection and confounding biases) in reducing disruptive behavior, level III/IV infractions, and school suspensions among at-risk children.

## Abbreviations

CICO: Check-In Check-Out
SCSD: Syracuse City School District
YF: Youth-First program
